# Supplementary figures and images for: Conflict resolution in socially housed Sumatran orangutans (Pongo abelii)
Source: PeerJ. 2018 Jul 31;6:e5303. doi: 10.7717/peerj.5303 (PMC6074800; doi:10.7717/peerj.5303)

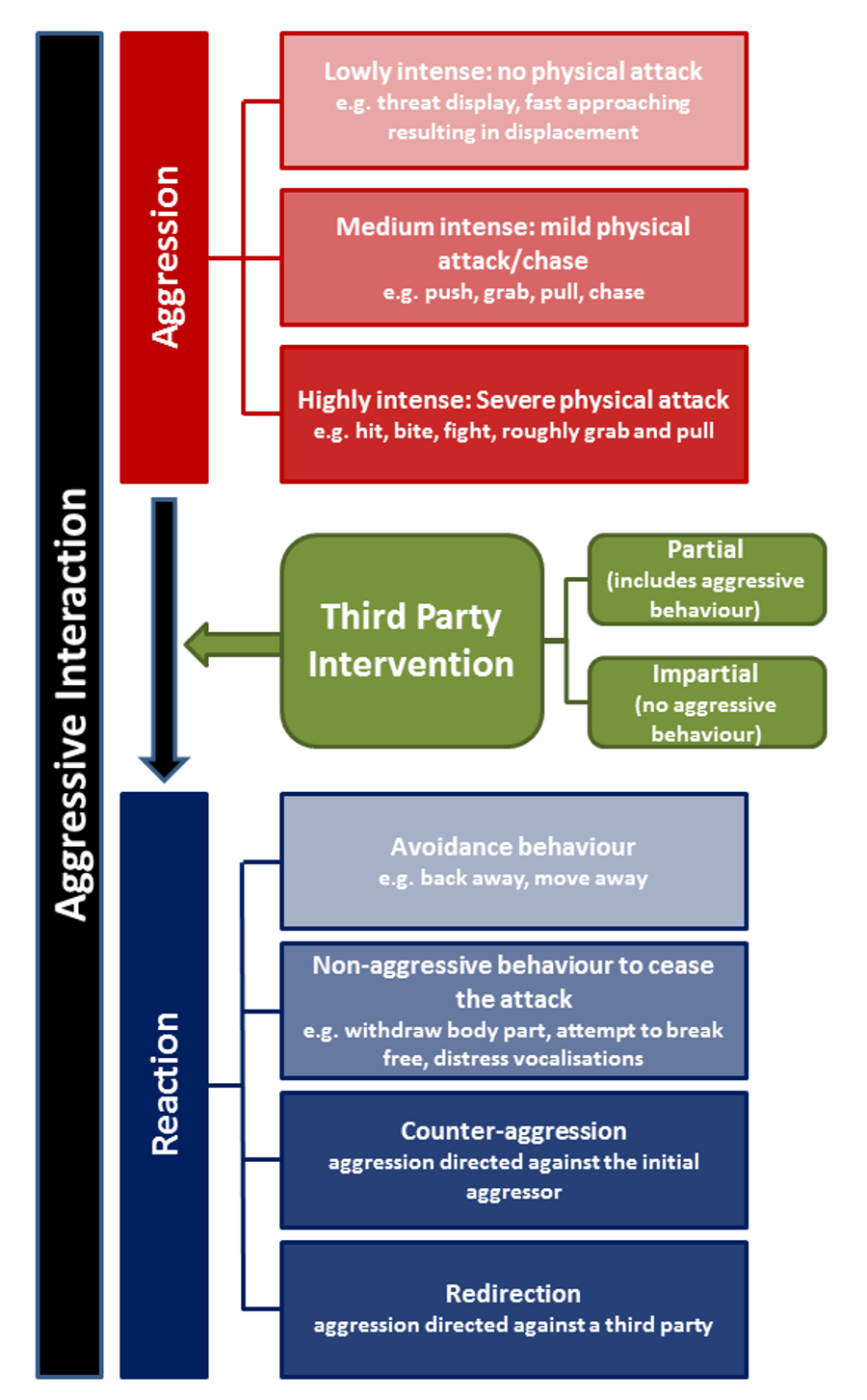

Supplement: Figure S1 [file peerj-06-5303-s001.png]

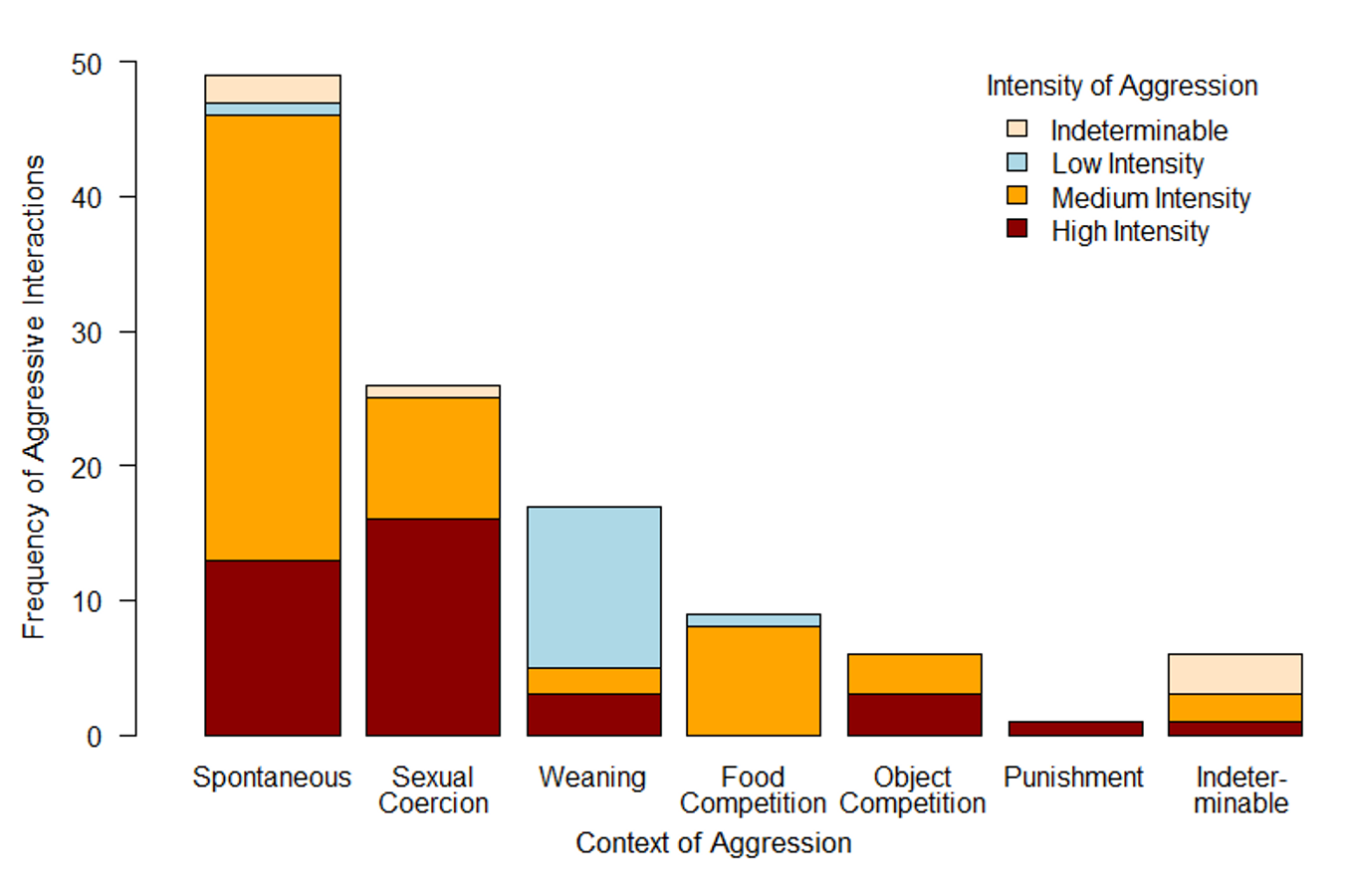

Supplement: Figure S2 — Each column indicates a specific context; different shades indicate different intensities of aggression. While spontaneous aggressions as the most frequent open conflicts were predominantly of medium intensity, the context of sexual coercion (note: this bar includes both male sexual coercion and sexual coercion among females) demonstrated a high proportion of highly intense aggressive interactions. On the contrary, most weaning conflicts were of low intensity. Open conflicts in the contexts of food or object competition or punishment played only a minor role. [file peerj-06-5303-s002.png]

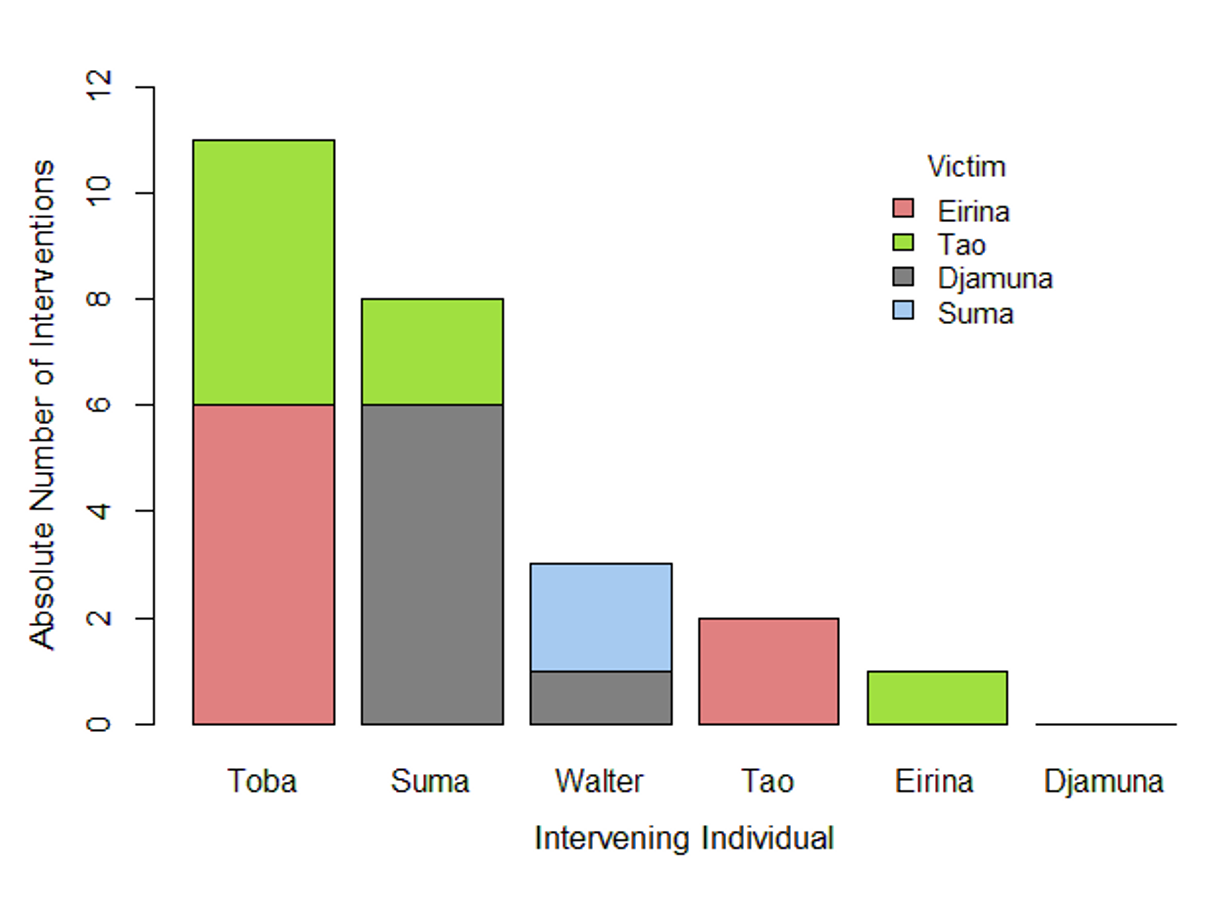

Supplement: Figure S3 — Each bar refers to a particular individual. Different colours indicate the particular victims involved in the respective intervened conflicts. [file peerj-06-5303-s003.png]
